# Cost-Effectiveness Analysis of Sequential Treatment Strategies for Advanced Melanoma in Real Life in France

**DOI:** 10.3390/curroncol29120725

**Published:** 2022-11-27

**Authors:** Marguerite Kandel, Aurélie Bardet, Stéphane Dalle, Clara Allayous, Laurent Mortier, Bernard Guillot, Caroline Dutriaux, Marie-Thérèse Leccia, Sophie Dalac, Henri Montaudie, Philippe Saiag, Delphine Legoupil, Florence Brunet-Possenti, Jean-Philippe Arnault, Julie De Quatrebarbes, Marie Beylot-Barry, Eve Maubec, Thierry Lesimple, François Aubin, Jean-Jacques Grob, Florence Granel-Brocard, Pierre-Emmanuel Stoebner, Alain Dupuy, Brigitte Dreno, Stefan Michiels, Céleste Lebbe, Isabelle Borget

**Affiliations:** 1 Department of Biostatistics and Epidemiology, Gustave Roussy, Paris-Saclay University, 94800 Villejuif, France; 2 Oncostat-U1018, Inserm, Paris-Saclay University, “Ligue Contre le Cancer” Labeled Team, 94800 Villejuif, France; 3 Dermatology, Hospices Civils de Lyon Hospital, Cancer Research Center of Lyon, Claude Bernard University, 69100 Lyon, France; 4 Department of Dermatology, Université de Paris, DMU ICARE, AP-HP Hôpital Saint Louis, 75010 Paris, France; 5 INSERM U976 HIPI, Team 1, F-75010 Paris, France; 6 CHRU Lille, INSERM, Lille University, U1189 Lille, France; 7 Dermatology, Montpellier Hospital, 34000 Montpellier, France; 8 Dermatology, Bordeaux Saint-André Hospital, 33000 Bordeaux, France; 9 Dermatology, Grenoble Hospital, 38000 Grenoble, France; 10 Dermatology, Dijon Hospital, 21000 Dijon, France; 11 Dermatology, Nice Hospital, 06000 Nice, France; 12 Dermatology, Assistance Publique des Hôpitaux de Paris, Ambroise Paré Hospital, 92100 Boulogne-Billancourt, France; 13 Dermatology, Brest Hospital, 29200 Brest, France; 14 Dermatology, Assistance Publique des Hôpitaux de Paris, Bichat Hospital, 75018 Paris, France; 15 Dermatology, Amiens Hospital, 80000 Amiens, France; 16 Dermatology, Annecy Hospital, 74370 Annecy, France; 17 Dermatology, Bordeaux Haut-L’évêque Hospital, 33075 Bordeaux, France; 18 Dermatology, Assistance Publique des Hôpitaux de Paris, Avicennes Hospital, 75004 Paris, France; 19 CLCC Rennes Eugène Marquis, 35000 Rennes, France; 20 Dermatology, Besançon Hospital, 25000 Besancon, France; 21 Dermatology, La Timone Hospital, 13005 Marseilles, France; 22 Dermatology, Nancy Hospital, 54000 Nancy, France; 23 Dermatology, Nîmes Hospital, 30900 Nimes, France; 24 Dermatology, Rennes Hospital, 35033 Rennes, France; 25 Dermatology, Nantes Hospital, 44200 Nantes, France; 26 GRADES Team, University Paris-Saclay, 91400 Saclay, France

**Keywords:** cost-effectiveness analysis, advanced melanoma, real-life clinical practice, immunotherapy, targeted therapy

## Abstract

Nine drugs have been marketed for 10 years for the treatment of advanced melanoma (AM). With half of patients reaching a second line, the optimal sequence of treatments remains unclear. To inform policy-makers about their efficiency, we performed a cost-effectiveness analysis of sequential strategies in clinical practice in France, for BRAF-mutated and wild-type patients. A multistate model was developed to describe treatment sequences, associated costs, and health outcomes over 10 years. Sequences, clinical outcomes, utility scores, and economic data were extracted from the prospective Melbase cohort, collecting individual data in 1518 patients since 2013, from their AM diagnosis until their death. To adjust the differences in patients’ characteristics among sequences, weighting by inverse probability was used. In the BRAF-mutated population, the MONO-targeted therapies (TT)-anti-PD1 sequence was the less expensive, whereas the anti-PD1-BI-TT sequence had an incremental cost-effectiveness ratio (ICER) of 180,441 EUR/QALY. Regarding the BRAF wild-type population, the three sequences constituted the cost-effective frontier, with ICERs ranging from 116 to 806,000 EUR/QALY. For BRAF-mutated patients, the sequence anti-PD1-BI-TT appeared to be the most efficient one in BRAF-mutated AM patients until 2018. Regarding the BRAF wild-type population until 2018, the sequence starting with IPI+NIVO appeared inefficient compared to anti-PD1, considering the extra cost for the QALY gained.

## 1. Introduction

Since 2011, nine novel drugs have been approved for the treatment of advanced melanoma (AM): immune-oncology therapies include ipilimumab (IPI, a CTLA-4 inhibitor), nivolumab, and pembrolizumab (NIVO and PEM, respectively, two PD-1 inhibitors) and the combination of NIVO+IPI. Targeted therapies (TT) approved for patients with BRAF mutation include monotherapy BRAF inhibitors (vemurafenib and dabrafenib, as MONO-TT), and combinations of bi-targeted therapies (BRAF+MEK inhibitors: vemurafenib+cobimetinib, dabrafenib+trametinib, and encorafenib+binimetinib, BI-TT). The overall survival (OS) of patients has significantly improved according to the results of clinical trials [1,2,3,4,5,6,7,8]. These therapeutics have also introduced new toxicities which question their impact on patients’ quality of life (QoL). QoL studies, mostly conducted in parallel with clinical trials, have shown a preserved QoL during the treatments [9,10,11,12,13,14,15]. Based on their clinical benefit, these drugs are granted high prices, up to 1000-fold higher than cytotoxic chemotherapies (CHEMO).

With a greatly increased survival rate and a preserved QoL, but with significant cost, several studies have assessed the efficiency of AM treatments. In the first line, immunotherapies are likely to be cost-effective [16,17,18,19,20,21], whereas targeted therapies are unlikely to be [22,23,24,25].

Considering the availability of multiple drugs, and with half of patients now receiving a second line of treatment, there is a need to identify the most efficient sequencing strategies. In BRAF-wild-type (wt) patients, cost-effectiveness analyses show discordant results, as Kohn et al. [17] identify PEM followed by IPI as the most cost-effective sequence, whereas it was the anti-PD1+IPI followed by chemotherapy sequence for Tarhini et al. [26]. In BRAF-mutated (m) patients, the only study available shows that a first line starting with anti-PD1+IPI is cost-effective, compared to a first line starting with bi-TT or anti-PD1 monotherapy [27]. Nevertheless, all these studies are based on models using data and comparators available in clinical trials, where patients are not necessarily representative of how they would be in real-life conditions [28]. In addition, except Wu et al. [29], who perform a cost-utility analysis in unknown BRAF status patients, none of these studies report cost-effectiveness results for either BRAF-wt or BRAF-m populations.

MelBase is a French prospective cohort enrolling patients with AM from 26 hospitals since 2013. The collected data in the ~1700 patients, including clinical outcomes, treatment lines, QoL and resource consumption, from their AM diagnosis until their death, allowed the first cost-effectiveness analysis using only individual patient data on treatment sequences that were used in clinical practice to treat a whole population of AM patients, reflecting real-life conditions.

The objective of this study was to perform a cost-effectiveness analysis of sequential strategies used for the treatment of AM in French clinical practice from 2013 to 2018, for BRAF-m and BRAF-wt patients.

## 2. Materials and Methods

### 2.1. Treatment Sequence Modelling

**Figure 1:**
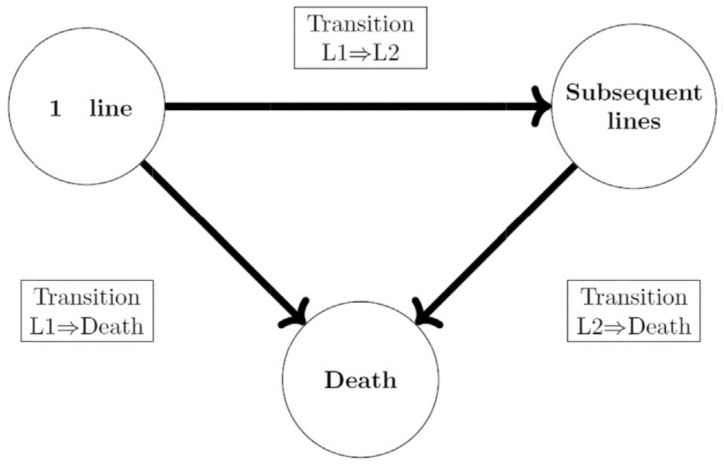
Multistate model. Legend: in such a model, the course of the disease is described using exclusive and exhaustive health states. The state occurrence and occupancy time depend on patients’ characteristics and time already spent in the present and previous states, meaning that the probability of moving to other states is influenced by the patient’s history and characteristics. In our case, the MSM described the individual patient’s treatment course (i.e., sequence) after their diagnosis of AM, with three states: “first line of treatment”, “subsequent lines of treatment”, and “death”.

A multistate model (MSM) [30], previously described [31], was developed to describe the treatment sequences, the associated total costs, and health outcomes over a time horizon of 10 years. Our MSM had three states: “first line of treatment”, “subsequent lines of treatment”, and “death”. All the patients entered into the “first line of treatment” state, and then moved into the “subsequent lines of treatment” state when they received a second line, or “death” when they died. Patients receiving a second line or more stayed in the “subsequent lines of treatment” state until death (Figure 1).

The treatment sequences to be compared have been identified from the MelBase cohort, reflecting the treatments approved or available in French clinical trials from 2013 to 2018. Unusual treatments or sequences involving fewer than 30 patients were excluded because they were not considered as representative of patient management at this period. Five sequences for the first and subsequent line of treatment of the BRAF-m population were included: Anti-PD1 → BI-TT, BI-TT → Anti-PD1, IPI+NIVO → BI-TT, MONO-TT → BI-TT and MONO-TT → Anti-PD1. For the BRAF-wt population, the comparison is based on the treatments received in the first line, regardless of the second line. Indeed, considering the great evolution of patient management between 2013 and 2018, a great heterogeneity shows up between receiving a second line. It creates multiple sequences with limited numbers of patients, generating a lack of robustness to perform the analyses. The strategies compared were: anti-PD1, IPI+NIVO, IPI alone and CHEMO. The flow-charts and sequences are presented in Figures S1 and S2.

### 2.2. Efficacy 

An MSM allows a description of the individual treatment course using exclusive and exhaustive health states. In MSMs, the state occupancy time depends on patients’ prognostic factors and the time already spent in the present and past states, meaning that the probability of moving to other states is influenced by the patient’s history. The MSM is based on regression models where each transition from one state to the other directly uses individual patient data from the MelBase cohort. For each sequence, the probabilities of staying in or moving from each state were derived from the associated Kaplan-Meier (KM) curves, estimated in MelBase patients. After that, KM curves were extrapolated over a time horizon of 10 years, as previously described [31].

### 2.3. Data

Patient characteristics, as well as their clinical outcomes, utility score, and economic data, were extracted from the MelBase cohort (NCT02828202) to generate the results in September 2018. MelBase is a prospective cohort, enrolling adults with AM at the time of AM diagnosis from 26 French hospitals. Enrolment in MelBase requires the availability of a tumor sample for histologic confirmation of advanced primary melanoma (unresectable stage III or stage IV), diagnosed at age 18 years or older, without prior systemic treatment other than adjuvant treatment. Patients with uveal melanoma and earlier stages of melanoma are not included in MelBase [32].

The 26 hospitals were selected based on their expertise in the treatment of MM, their infrastructure (a biobank was mandatory) and their will to participate. Data collection per patient was prospective through an electronic questionnaire. MelBase is sponsored by the French National Cancer Institute and industrial partners and is administered by the Parisian Public Hospitals (AP-HP) Department of Clinical Research and Innovation. The MelBase protocol was approved by the French Ethics Committee. Written informed consent was obtained from all patients.

To adjust the differences in patient characteristics and confounding effects among treatment sequences in this observational context, patients were weighted using the inverse of probability treatment (IPTW). The probability of treatment was estimated using a propensity score (PS) based on a multivariate model. The PS is a quantitative value that summarizes the initial patients’ characteristics to form comparable groups that differ only by the treatment actually received. The PS for multiple treatments was estimated using generalized boosted modeling (GBM) [33] implemented by the twang [34] package in R with ten baseline variables. These 10 variables (presence of brain or liver metastases, elevated LDH, ECOG score, melanoma metastatic stage, elevated neutrophil/lymphocyte ratio, mutation BRAF V600E, age > 65 years, anatomic location of melanoma, body-mass index) were selected because they are known to have a prognostic role in survival. The distributions of the variables across the different sequences were compared before and after weighting (see Tables S1–S4 for BRAF-m and BRAF-wt populations, respectively).

### 2.4. Costs of Care

Resource uses were prospectively collected per patient in MelBase cohort, from MM diagnosis to death or the last follow-up. Valued data includes treatments (reimbursed drugs, except drugs prescribed into a clinical trial, and administration), hospitalizations (including toxicity and surgery), radiotherapy sessions, biological and radiological exams and visits. Costs were assessed from the French health insurance perspective and were indicated to be EUR 2019 [31]. A monthly cost per treatment and per line was reported.

### 2.5. Utility Estimates

Utility scores per line and per treatment were obtained from individual patient data collected in the MelBase cohort using the three-level EuroQol five-dimensional (EQ-5D 3L) questionnaire. Questionnaires were filled out by patients themselves and assessed at inclusion, every 3 months and at each change of melanoma therapy, until death. QoL was analyzed for all patients who had at least one complete questionnaire, by the line of each treatment sequence. Responses were converted into a utility index [35] using the French value set [36]. To assess longitudinal QoL evolution across first-line treatment and subsequent lines, mixed-effects models for repeated measures (MMRM) were used, as previously described [37].

### 2.6. Analyses

The time spent in each state (line), total cost, and quality-adjusted life-years (QALYs) were calculated by the area under each KM curve. Costs and QALYs were discounted at a rate of 4%, as recommended in France [38] in 2019.

Confidence intervals (CI) for cost and QALY were obtained using empirical percentiles from non-parametric bootstrapping (1000 replicates).

All models (MSM, GBM, and MMRM) were implemented using R software. However, the cost-effectiveness analysis required adapting the code from the tutorial of Williams et al. [39,40] to incorporate the propensity score obtained by GBM, by taking into account the adjustment on multiple covariates. To evaluate the robustness of our analysis, sensitivity analyses were performed by fluctuating the time horizon (5 and 15 years) and the discount rate at 0%.

## 3. Results

### 3.1. Cohort Characteristics

Between March 2013 and September 2018, 1719 patients were included in the MelBase cohort. Among them, patients were excluded because their follow-up was less than 6 months (*n* = 117), they did not receive a systemic treatment (*n* = 34), or did not meet the inclusion criteria from the Melbase cohort (*n* = 50). Among the remaining 1518 patients, the mean age was 64 years, 58% were men and 42% were BRAF-mutated. On the 3rd January 2019 (point date), the median follow-up was 13.9 months (range: 0.1–68.9), and 52% of patients had died. Table 1 summarizes the patients’ characteristics and treatments.

### 3.2. Cost and Utility per Line

Table 2 shows the monthly cost of management per patient, and utility score per treatment, as observed in the MelBase cohort. Whatever the line or the BRAF status, sequences with IPI+NIVO and BI-TT were the most expensive, as the drug represented 80% of the total cost. Utility scores were similar between treatments.

### 3.3. Survival, QALY and Cost over 10 Years

For each sequence, the estimated time spent in first line and in subsequent-lines, total life-months, QALYs, and total cost are presented in Table 3, as estimated by the MSM models. Whatever the BRAF status, results showed a high level of heterogeneity, making the interpretation complicated.

Regarding BRAF-m patients, the Anti-PD1 → BI-TT sequence seemed to have the greatest efficacy, both in terms of survival time and QALY. In the BRAF-wt population, the 4 sequences showed similar results in terms of QALYs, but the IPI+NIVO sequence was the most expensive with a total 10-year cost of EUR 351,590 [_95%_CI: 223,829–517,784].

### 3.4. Cost-Effectiveness Analysis

**Figure 2:**
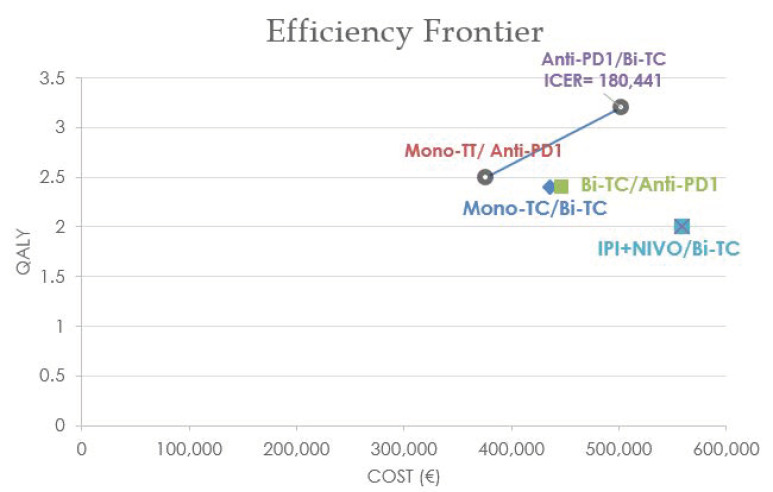
Efficiency frontier for the BRAF-mutated population.

In the BRAF-m population, by incrementally examining the sequences, starting from the less expensive (MONO-TT → antiPD1), three sequences were excluded from the efficiency frontier (Figure 2) as they were more expensive and less effective than this last sequence. The anti-PD1 → BI-TT sequence was the only sequence remaining on the efficiency frontier, with 0.7 QALYs gained for an extra cost of EUR 126,309, resulting in an incremental cost-effectiveness ratio (ICER) of 180,441 EUR/QALY.

**Figure 3:**
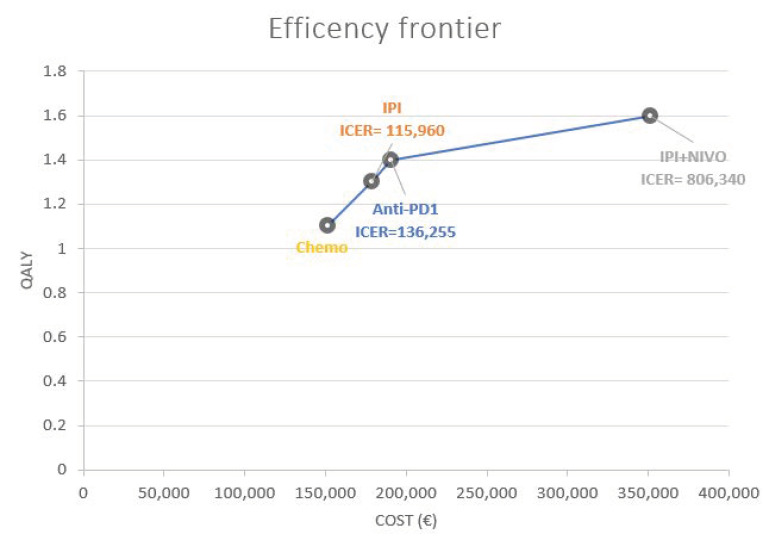
Efficiency frontier for the BRAF wild-type population.

Concerning the BRAF-wt population, starting from the CHEMO sequence (the less expensive), the three other sequences constituted the cost-effective frontier (Figure 3), as they generated a gain on QALY for an additional cost. Thus, the ICERs were respectively 136,255 EUR/QALY for Anti-PD1 compared to Chemotherapy, 115,960 EUR/QALY for Ipilimumab compared to Anti-PD1 and 806,340 EUR/QALY for Ipilimumab-Nivolumab compared to Ipilimumab. 

### 3.5. Sensitivity Analyses

By varying time the horizon (5 and 15 years) and the discount rate (0%), the results remained consistent with the base case: efficiency frontier remained unchanged, ICER and acceptability curves were similar, showing the robustness of our results (Supplementary Materials).

## 4. Discussion

With the increasing number of treatment options and the improved outcomes of these innovative drugs, most patients with AM now receive multiple lines of therapy. The prolonged survival time of patients, as well as the use of drug associations, has a considerable economic impact on the management of AM. The proof is, the average cost per patient in France has increased from EUR 1634 in 2004 to almost EUR 270,000 in 2017 [31]. Although various studies have already assessed the efficiency of first-line treatment, determining the optimal therapeutic sequence has become the biggest challenge for clinicians, patients, and payers. Few cost-effectiveness analyses have explored sequential treatments and ended up providing discordant results. This work is the first to perform a cost-effectiveness analysis comparing sequential strategies used for the treatment of AM in real life in France up to 2018, for both BRAF-m and BRAF-wt patients.

Our analysis demonstrated that, among the BRAF-m population, only two sequences (Anti-PD1 → Bi-TT and MONO-TT → Anti-PD1) were present on the efficiency frontier. These results are in accordance with previous studies, showing that anti-PD1 were found to be the most cost-effective sequences. We also showed that the three other sequences, which all included a bi-TT, were excluded from the frontier efficiency because of their higher costs, which is in line with the previous results observed. Nevertheless, contrary to Tahrari [26] and Wu [29], who concluded that IPI+NIVO maximizes health outcomes, we observed that this sequence was associated with similar survival gain but with higher cost, making this strategy apparently not cost-effective in the French context up to 2018. This needs to be re-evaluated when the combination IPI+NIVO will have facilitated access by French authorities.

In the BRAF-wt population, we observed that all three sequences starting with anti-PD1, IPI alone, or IPI+NIVO were more effective but also more expensive than chemotherapy, being then on the efficiency frontier. These results were also in accordance with the previous studies, except for the IPI alone strategy, which was excluded from the efficiency frontier by Kohn [17] and Tarhini [27], because of its high cost. Even if it seems that IPI+NIVO was the most effective among the BRAF-wt population, we observed, similar to the other studies, that it was the least cost-effective strategy, as it is almost twice more expensive. With an ICER estimated at 806,000 EUR/QALY, this sequence cannot be considered cost-effective in the French context.

Our study is the first to assess efficiency using individual patient data from the MelBase cohort, the largest French cohort of AM patients, and guaranteeing a good representativity by integrating 65% of the French patients with systemic treatment. This provides many advantages. First, it reflected real-life conditions and non-selected patients. Secondly, given the large size of the MelBase cohort, it enables the inclusion of multiple comparators, whereas previous studies were restricted to the comparators available in clinical trials. The sequences compared in our study were clinically relevant and representative of the treatment used in France from 2013 to 2018. Third, it permits conducting a cost-effectiveness analysis both on BRAF-m and BRAF-wt populations. Nevertheless, the observational context of the MelBase cohort generates some methodological challenges. While most cost-effectiveness studies use aggregated data from clinical trials, we developed an MSM, i.e., a patient-level model, that considers patients’ characteristics and their history to describe their treatment course. Subsequently, an advanced methodology was used to make the patients comparable, through individually weighted patient data. The use of these methods, based on observational and individual data, will undoubtedly increase in the future, considering the great number of innovative drug marketing with increased survival and a short follow-up time of clinical trials.

This study has some limitations. First, the maturity of the efficacy data differs between treatments, with the median OS not being reached for all treatments, extrapolation is therefore based on high uncertainty. Furthermore, France does not reimburse IPI+NIVO in BRAF-m patients, introducing a selection bias for these patients, which probably plays against IPI+NIVO, more than that the sequence of treatment in patients with BRAF-m AM is changing and may affect costs in the near future [41]. However, our results are not modified in the various sensitivity analyses. Then, from a methodological point of view, although inverse probability weighting has made it possible to consider imbalances of measured confounding covariates to a certain degree, only randomized clinical trials can account for possible imbalances in unmeasured confounders. Indeed, the economic benefits of immunotherapy treatment on society (such as loss of productivity) were not taken into account, as it is not recommended in France, and they were not collected directly in the MelBase cohort. Finally, it is important to put these results into perspective with respect to the evolution of the management of patients with AM between 2013 and 2018. This work integrates comparators that are to date rarely used, as chemotherapy in first line. This point can be explained by three elements. The first is the rapid evolution of patient care, including treatment sequences that were initiated before the stabilization of MM management. Secondly, according to the recommendations of the French Health Authority, an economic evaluation analysis needs to include all available treatments, and a limited or previous use of a treatment is not a sufficient argument to justify its exclusion. Indeed, the non-inclusion of chemotherapies would have generated uncertainty about the ranking of these strategies regarding their efficiency. Finally, these innovative treatments reveal a learning curve for clinicians, as the management of severe toxicity of immunotherapy combinations was not the same in 2017 as it is today, therefore potentially reducing efficacy.

## 5. Conclusions

In conclusion, in the BRAF-m population from 2013 to 2018, the most efficient sequence appeared to be Anti-PD1 → BI-TT based on the different analyses without the possibility of really assessing sequences involving IPI+NIVO. Regarding wild-type MM patients, our estimations suggest that the IPI+NIVO strategy may not be efficient with a cost that is too high with regards to the effectiveness gained (keeping in mind the same complicated access to this combination in France). 

## Figures and Tables

**Table 1:** Initial characteristics and treatments received by the 1518 patients of the MelBase cohort, for the whole cohort and according to their BRAF status.

Characteristics	MelBase Patients (*n* = 1518), No. (%) or Mean (Range)	BRAF-Mutated Patients (*n* = 639), No. (%) or Mean (Range)	BRAF-Wild-Type Patients (*n* = 879), No. (%) or Mean (Range)
**Age (years)**	64 (18–98)	58.3 (18–94)	67.6 (25–98)
<65 years	719 (47.4)	406 (63.5)	313 (35.6)
≥65 years	799 (52.6)	233 (36.5)	566 (64.4)
**Gender**			
Male	879 (57.9)	368 (57.6)	511 (58.1)
Female	639 (42.1)	271 (42.4)	368 (41.9)
**Years of inclusion**			
2013	132 (8.7)	50 (7.8)	82 (9.3)
2014	267 (17.6)	115 (18.0)	152 (17.3)
2015	338 (22.2)	147 (23.0)	191 (21.7)
2016	332 (21.9)	160 (25.0)	172 (19.6)
2017	305 (20.1)	111 (17.4)	194 (22.1)
2018 ^1^	144 (9.5)	56 (8.8)	88 (10.0)
**BRAF Mutated**	639 (42.1)	639 (100)	0
**V600E**	498 (78.3)	498 (78.3)	
**Wild-type**	879 (57.9)	0	879 (100)
**Elevated LDH > 1 ULN ^2^**	461 (30.4)	197 (30.9)	264 (30.1)
**High neutrophil to lymphocyte ratio**	430 (28.3)	196 (30.7)	234 (27.1)
**Presence of metastases**			
Brain	272 (17.9)	146 (22.9)	126 (14.3)
Liver	413 (27.2)	182 (28.5)	231(26.3)
**ECOG performance status**			
0,1	1248 (82.2)	520 (81.4)	728 (82.8)
2,3,4	116 (7.6)	49 (7.7)	67 (7.6)
Unknown	154 (10.1)	70 (11.0)	84 (9.6)
**Melanoma Metastatic status**			
Unresectable stage IIIC	154 (10.1)	46 (7.2)	108 (12.3)
M1a	145 (9.6)	64 (10.0)	81 (9.2)
M1b	251 (16.5)	75 (11.7)	176 (20.0)
M1c	891 (58.7)	422 (66.0)	469 (53.4)
Unknown	77 (5.1%)	32 (5.0)	45 (5.1)
**Anatomic location of melanoma**			
Upper and lower extremities	440 (29.0)	171 (26.8)	269 (3.1)
Trunk	461 (30.4)	276 (43.2)	185 (21.0)
Head and Neck	210 (13.8)	85 (13.3)	125 (14.2)
Acral lentiginous	103 (6.8)	14 (2.2)	89 (10.1)
Mucosa	100 (6.6)	9 (1.4)	91 (10.4)
Unknown	204 (13.4)	84 (13.1)	120 (13.7)
**BMI ^3^**			
<30	1215 (80.0)	521 (81.5)	694 (79.0)
>30	303 (20.0)	118 (18.5)	185 (21.0)
**First line of treatment**			
Cytotoxic chemotherapy	88 (5.8)	5 (0.8)	83 (9.4)
Ipilimumab	202 (13.3)	16 (2.5)	186 (21.2)
Ipilimumab + Nivolumab	163 (10.7)	51 (8.0)	112 (12.7)
Anti-PD1	540 (35.6)	78 (12.2)	462 (52.6)
Mono-targeted therapy	182 (12.0)	155 (24.3)	27 (3.1)
Bi-targeted therapy	326 (21.5)	325 (50.9)	1 (0.1)
Included in a clinical trial	17 (1.1)	9 (1.4)	8 (0.9)
**Second line of treatment**			
None, as still on first line of treatment at data cut-off	318 (21.0)	128 (20.0)	190 (21.6)
None, as dead during the first line	299 (19.7)	88 (13.8)	211 (24.0)
Off treatment	108 (7.1)	20 (3.1)	88 (10.0)
**Second line observed including:**	793 (52.2)	403 (63.1)	390 (44.4)
**Chemotherapy**	107 (13.5)	24 (3.8)	83 (21.3)
**Ipilimumab**	143 (18.0)	15 (2.3)	128 (32.8)
**Ipilimumab + Nivolumab**	18 (2.3)	11 (1.7)	7 (1.8)
**Anti-PD1**	317 (40.0)	180 (28.2)	137 (35.1)
**Mono-targeted therapy**	67 (8.4)	51 (8.0)	16 (4.1)
**Bi-targeted therapy**	104 (13.1)	103 (16.1)	1 (0.3)
**Included in a clinical trial**	37 (4.7)	19 (3.0)	18 (4.6)

^1^ Until 08/31/2018, ^2^ ULN= upper limit of normal, ^3^ Body Mass Index.

**Table 2:** Monthly cost of management per patient and utility scores observed from the MelBase cohort, in the BRAF-m and BRAF-wt patients.

	BRAF-Mutated Patients					BRAF Wild-Type Patients			
First Line of Treatment (L1)	Anti-PD1	Bi-Targeted Therapy	Ipilimumab + Nivolumab	Mono-Targeted Therapy	Mono-Targeted Therapy	Anti-PD1	Ipilimumab + Nivolumab	Ipilimumab	Chemotherapy
Second Line of Treatment (L2)	Bi-Targeted Therapy	Anti-PD1	Bi-Targeted Therapy	Bi-Targeted Therapy	Anti-PD1				
**Cost/month in L1**	**7723 ± 3701**	**13,050 ± 6867**	**26,966 ± 32,824**	**11,932 ± 11,962**	**8165 ± 5077**	**6769 ± 4433**	**11,412 ± 11,994**	**15,858 ± 29,563**	**3381 ± 6996**
(mean ± SD ^1^, EUR)									
Drug	5730 ± 3194	12,107 ± 6518	22,023 ± 26,959	10,693 ± 11,675	7320 ± 4043	4839 ± 2567	9112 ± 10,111	13,708 ± 27,560	537 ± 5256
Administration	541 ± 159	0	1246 ± 947	0	0	546 ± 265	817 ± 498	401 ± 865	0
Radiotherapy	363 ± 1608	116 ± 358	331 ± 925	259 ± 944	123 ± 471	207 ± 1166	73 ± 240	231 ± 928	235 ± 1122
Biological exam	121 ± 70	54 ± 30	109 ± 68	84 ± 136	64 ± 55	119 ± 70	90 ± 62	146 ± 472	85 ± 104
Radiological exam	74 ± 50	76 ± 53	127 ± 74	150 ± 180	102 ± 100	73 ± 71	74 ± 79	130 ± 447	145 ± 215
Hospitalization	1334 ± 1925	1104 ± 1139	4768 ± 6278	954 ± 1109	1204 ± 1986	1444 ± 3032	1715 ± 3002	1653 ± 2453	3220 ± 7448
Visit	28 ± 12	15 ± 8	17 ± 12	25 ± 22	20 ± 15	32 ± 12	21 ± 12	22 ± 12	21 ± 25
**Cost/month in L2**	**11,127 ± 3361**	**8685 ± 5525**	**10,733 ± 3917**	**9566 ± 3731**	**9481 ± 13,063**	**7932 ± 5796**	**14,956 ± 40,275**	**4942 ± 2688**	**7551 ± 5256**
(mean ± SD ^1^, EUR)									
Drug	9804 ± 3484	6006 ± 4058	9498 ± 4163	8309 ± 3366	6004 ± 5099	6202 ± 5068	12,586 ± 37,246	3386 ± 2083	6458 ± 4915
Administration	209 ± 122	523 ± 338	140 ± 79	143 ± 97	433 ± 446	282 ± 193	2499 ± 4539	433 ± 236	357 ± 205
Radiotherapy	107 ± 347	193 ± 447	45 ± 99	53 ± 140	480 ± 1656	203 ± 518	142 ± 337	140 ± 304	35 ± 94
Biological exam	49 ± 22	101 ± 70	48 ± 24	45 ± 35	86 ± 48	88 ± 271	67 ± 51	100 ± 73	89 ± 55
Radiologic exam	46 ± 20	51 ± 41	53 ±38	50 ± 42	51 ± 28	44 ± 30	46 ± 21	57 ± 89	57 ± 51
Hospitalization	1275 ± 1621	2233 ± 3164	1084 ± 1184	1116 ± 1374	2638 ± 8473	1924 ± 2030	2884 ± 4543	1050 ± 1774	1068 ± 1145
Visit	22 ± 11	38 ± 20	18 ± 8	17 ± 9	34 ± 28	35 ± 84	75 ± 187	36 ± 36	29 ± 13
**Cost of palliative care** (mean ± SD ^1^, EUR)	2928 ± 5349	2477 ± 4329	1247 ± 2864	2652 ± 4922	2652 ± 4922	1713 ± 3037	1407 ± 3944	2900 ± 5562	2240 ± 3411
**Utility L1** (mean, CI_95%_^2^)	0.75 (0.65–0.85)	0.77 (0.72–0.81)	0.68 (0.57–0.80)	0.66 (0.57–0.75)	0.76 (0.68–0.84)	0.74 (0.71–0.76)	0.75 (0.70–0.80)	0.73 (0.68–0.78)	0.67 (0.59–0.75)
**Utility L2** (mean, CI_95%_^2^)	0.81 (0.72–0.90)	0.73 (0.69–0.77)	0.70 (0.60–0.79)	0.70 (0.62–0.78)	0.72 (0.65–0.80)	0.64 (0.60–0.68)	0.65 (0.57–0.74)	0.70 (0.65–0.74)	0.65 (0.56–0.73)

^1^ SD = Standard Deviation, ^2^ CI = Confidence Interval.

**Table 3:** Mean time spent in first and subsequent line of treatment, survival time, QALY, and total cost per patient from the multistate model (MSM) at 10 years.

	Sequence	Mean Time Spent in First Line ([CI_95%_], (Month, Years))	Mean Time Spent in Subsequent Lines ([CI_95%_], (Month, Years))	Mean Survival Time ([CI_95%_], (Month, Years))	Mean QALYs ^3^ [CI_95%_]	Mean Total Cost per Patient [CI_95%_], (EUR)
**BRAF-mutated patients**	Anti-PD1 → Bi-T ^2^	15.6 (5.7–25.8) 1.3 (0.5–2.1)	32.3 (13.4–59.7) 2.7 (1.1–5.0)	47.9 (25.6–73.6) 4.0 (2.1–6.1)	3.2 (1.7–4.9)	502,045 (262,525–787,754)
	Bi-TT ^2^ → Anti-PD1	21.4 (14.1–32.0) 1.8 (1.2–2.7)	17.0 (7.3–26.4) 1.4 (0.6–2.2)	38.4 (26.1–51.7) 3.2 (2.2–4.3)	2.4 (1.6–3.2)	446,766 (313,691–608,496)
	IPI+NIVO → Bi-TT ^2^	9.7 (4.4–16.5) 0.8 (0.4–1.4)	25.7 (13.2–43.6) 2.1 (1.1–3.7)	35.4 (20.9–54.9) 2.9 (1.7–4.6)	2.0 (1.2–3.2)	558,168 (343,439–813,136)
	MONO-TT ^1^ → Bi-TT ^2^	12.1 (7.0–25.6) 1.0 (0.6–2.1)	29.4 (12.2–45.2) 2.5 (1.0–3.8)	41.5 (23.1–62.4) 3.5 (1.9–5.2)	2.4 (1.3–3.6)	435,702 (246,112–663,460)
	MONO-TT ^1^ → Anti-PD1	21.4 (13.2–38.6) 1.8 (1.1–3.2)	18.9 (7.0–37.2) 1.6 (0.6–3.1)	40.3 (26.1–64.8) 3.4 (2.2–5.4)	2.5 (1.6–4.0)	375,736 (240,740–604,066)
**BRAF Wild-Type patients**	Anti-PD1	14.7 (10.6–17.9) 1.2 (0.9–1.5)	8.0 (4.9–12.2) 0.7 (0.4–1.0)	22.7 (17.0–27.5) 1.9 (1.4–2.3)	1.3 (1.0–1.6)	178,726 (132,270–218,287)
	Ipi + Nivo	21.3 (11.6–31.9) 1.8 (1.0–2.7)	5.3 (2.6–12.1) 0.4 (0.2–1.0)	26.6 (16.5–38.9) 2.2 (1.4–3.2)	1.6 (1.0–2.4)	351,590 (223,829–517,784)
	Ipilimumab	5.6 (3.9–8.7) 0.5 (0.3–0.7)	18.5 (10.0–27.1) 1.5 (0.8–2.3)	24.1 (15.9–32.7) 2.0 (1.3–2.7)	1.4 (0.9–1.9)	190,322 (137,526–247,713)
	Chemotherapy	4.1 (2.2–6.8) 0.3 (0.2–0.6)	16.3 (8.0–27.0) 1.4 (0.7–2.3)	20.4 (11.9–31.1) 1.7 (0.6–2.3)	1.1 (0.7–1.7)	151,475 (84,039–239,747)

^1^ MONO-TT = Mono-targeted therapy, ^2^ Bi-TT = Bi-targeted therapy, CI = Confidence Interval, ^3^ QALY = Quality adjusted life year.

## Data Availability

The data presented in this study are available on request from the corresponding author.

## Supplementary Materials

The following supporting information can be downloaded at: https://www.mdpi.com/article/10.3390/curroncol29120725/s1, Figure S1: Flow-chart of BRAF-mutated population from the MelBase cohort, Figure S2: Flow-chart of BRAF-wild-type population from the MelBase cohort, Figrue S3: Cost-effectiveness acceptability curve for BRAF population at 5 years, Figure S4: Cost-effectiveness acceptability curve for Wild-type population at 5 years, Figure S5: Cost-effectiveness acceptability curve for BRAF population at 15 years, Figure S6: Cost-effectiveness acceptability curve for Wild-type population at 15 years, Figure S7: Cost-effectiveness acceptability curve for BRAF population with a discount rate set at 0%, Figure S8: Cost-effectiveness acceptability curve for Wild-type population with a discount rate set at 0%, Table S1: Distribution of the baseline variables in BRAF-mutated population before weighting, Table S2: Distribution of baseline variables in BRAF-mutated population after weighting, Table S3: Distribution of baseline variables in BRAF Wild-type population before weighting, Table S4: Distribution of the baseline variables in BRAF-Wild-type population after weighting, Table S5: Extrapolated life expectancy and cost per patient from the multistate model (MSM) at 5 years, Table S6: Extrapolated life expectancy and cost per patient from the multistate model (MSM) at 15 years, Table S7: Extrapolated life expectancy and cost per patient from the multistate model (MSM) with a discount rate set at 0%.
