# Supplementary material for: Cost-Effectiveness Analysis of Sequential Treatment Strategies for Advanced Melanoma in Real Life in France
Source: Curr Oncol. 2022 Nov 27;29(12):9255–70. doi: 10.3390/curroncol29120725 (PMC9777106; doi:10.3390/curroncol29120725)
Supplement: Supplementary file 1 [file curroncol-29-00725-s001.zip › curroncol-2009213-supplementary.pdf]

Supplementary files

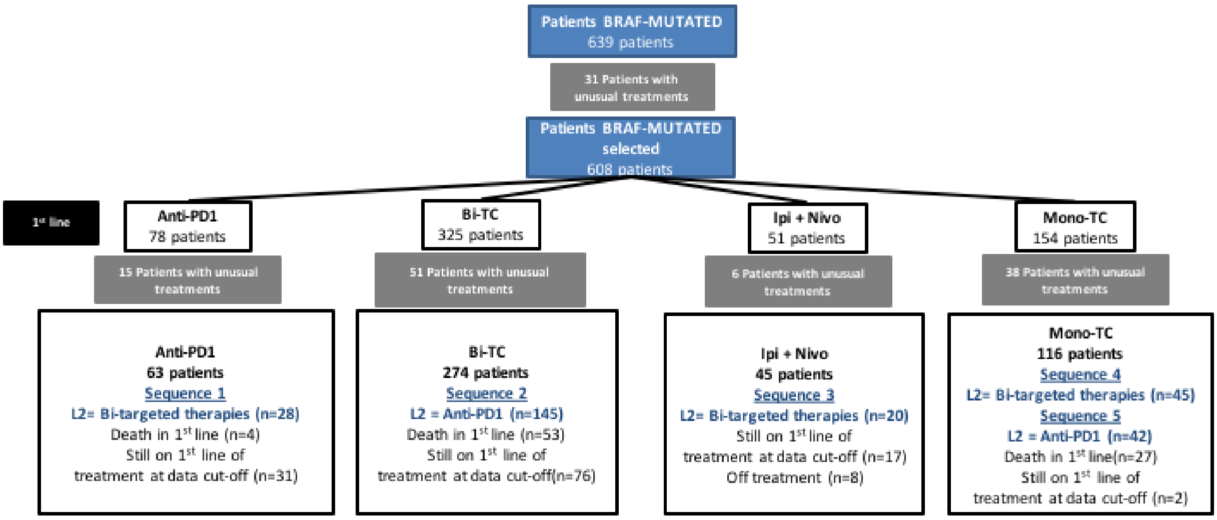

Figure S1: Flow-chart of BRAF-mutated population from the MelBase cohort

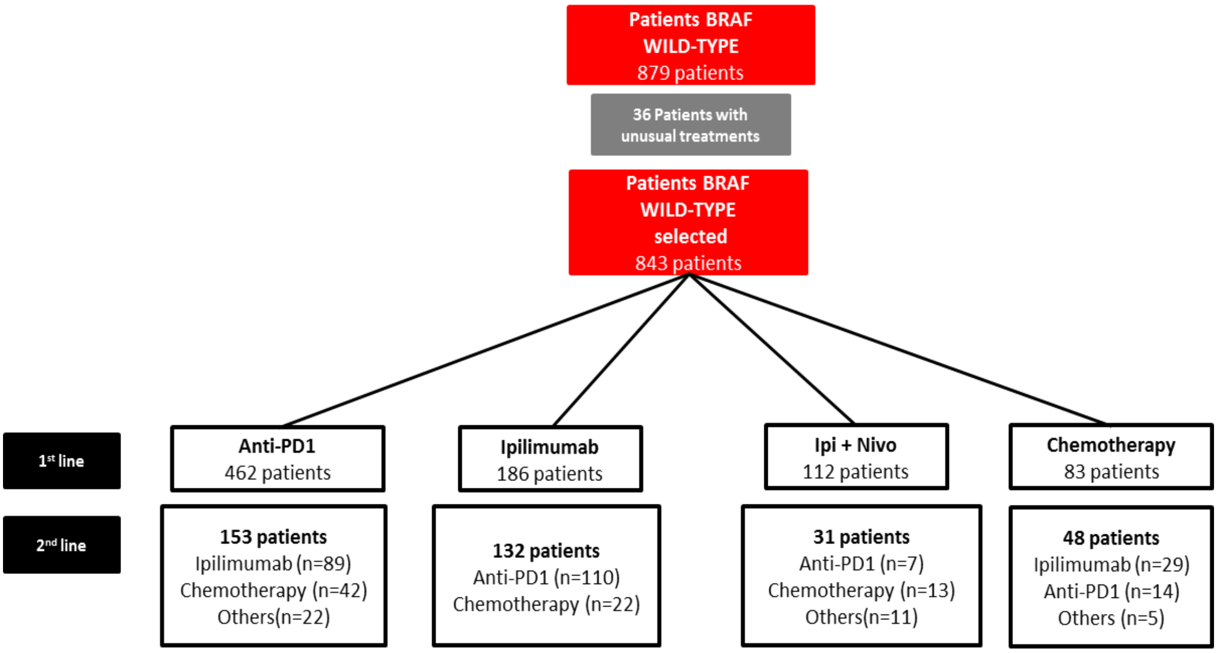

Figure S2: Flow-chart of BRAF-wild-type population from the MelBase cohort

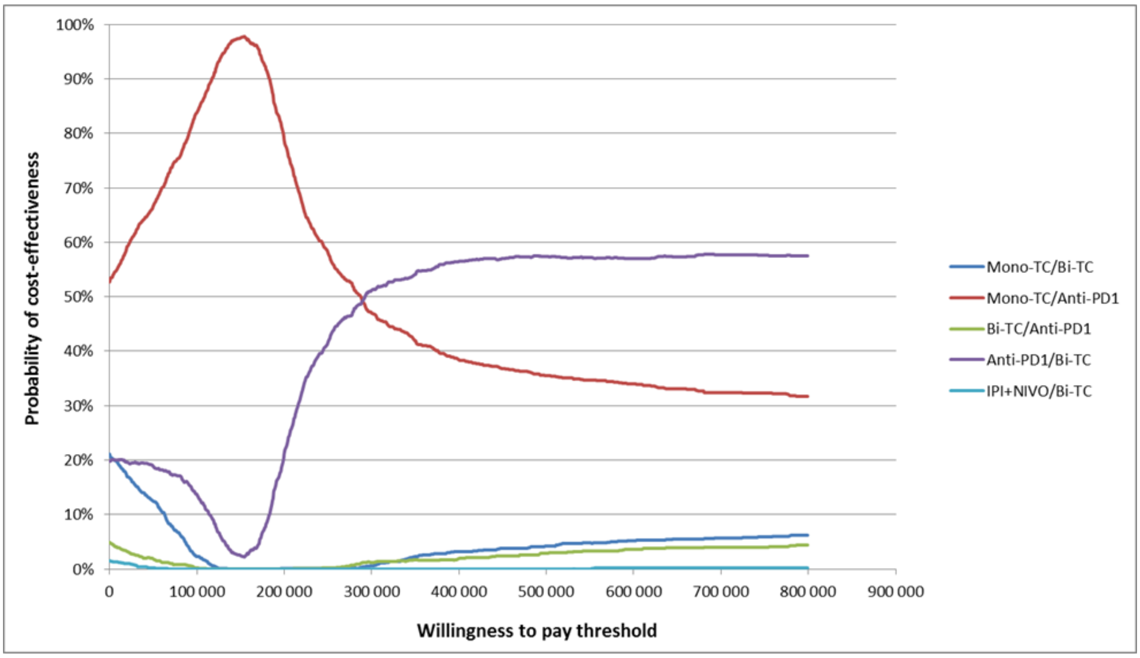

Figure S3: Cost-effectiveness acceptability curve for BRAF population at 5 years

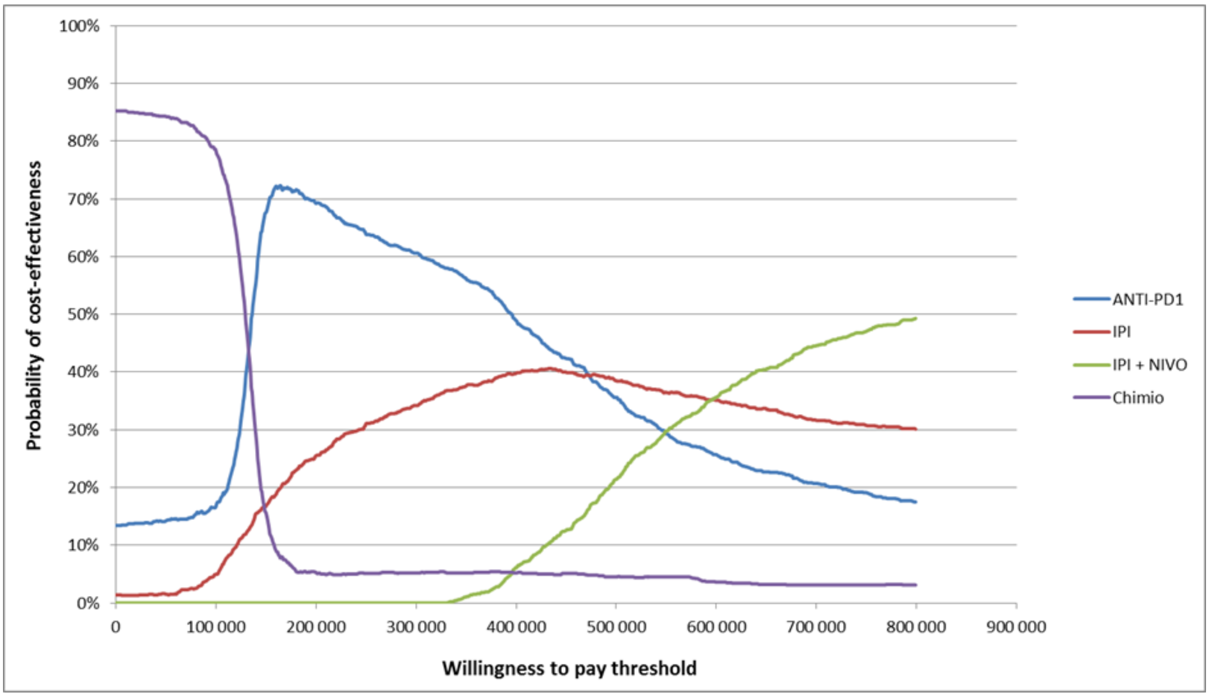

Figure S4: Cost-effectiveness acceptability curve for Wild-type population at 5 years

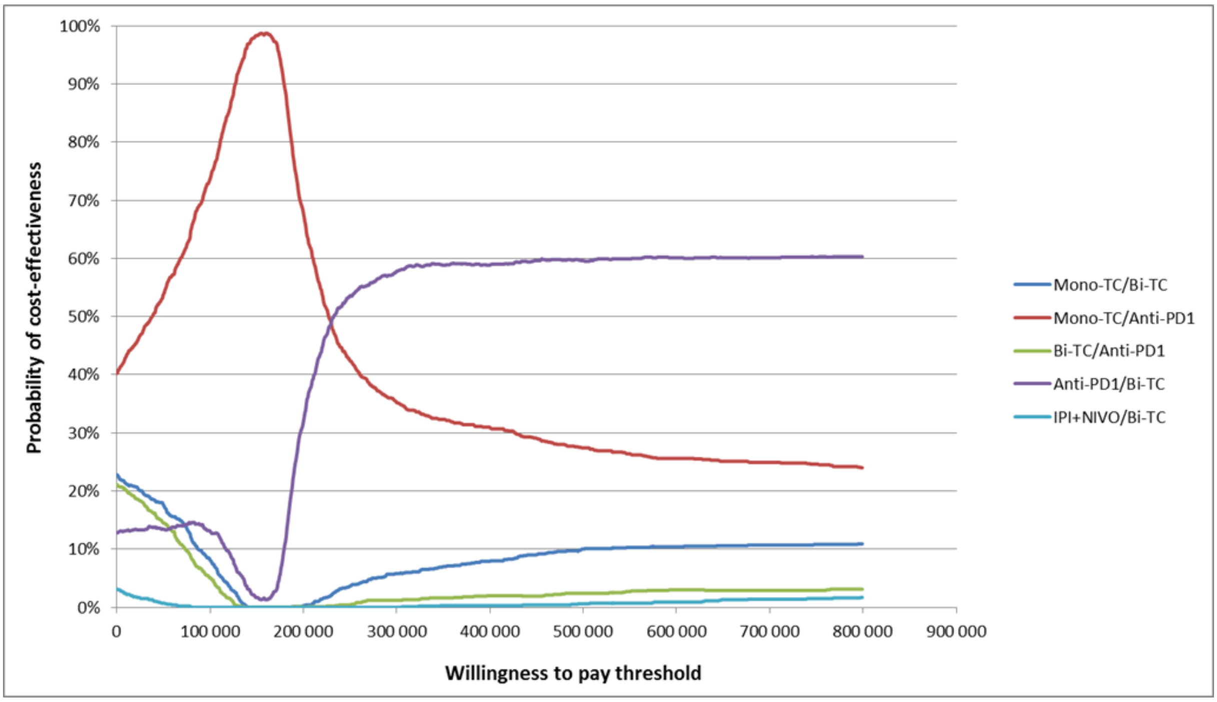

Figure S5: Cost-effectiveness acceptability curve for BRAF population at 15 years

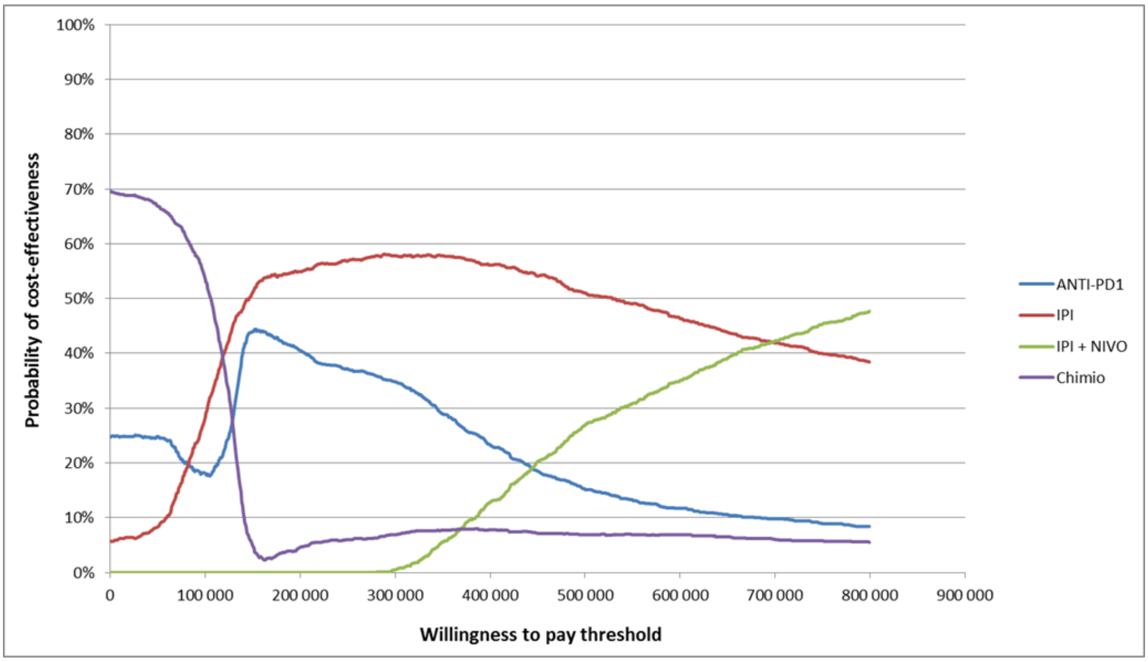

Figure S6: Cost-effectiveness acceptability curve for Wild-type population at 15 years

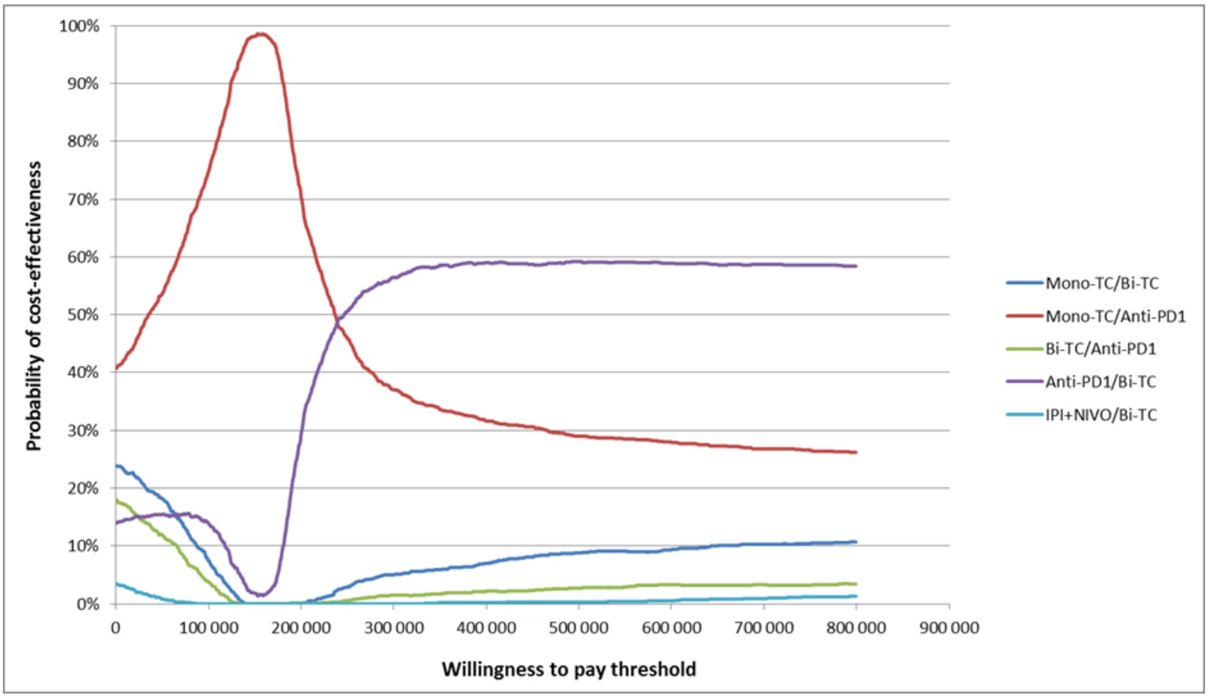

Figure S7: Cost-effectiveness acceptability curve for BRAF population with a discount rate set at 0%

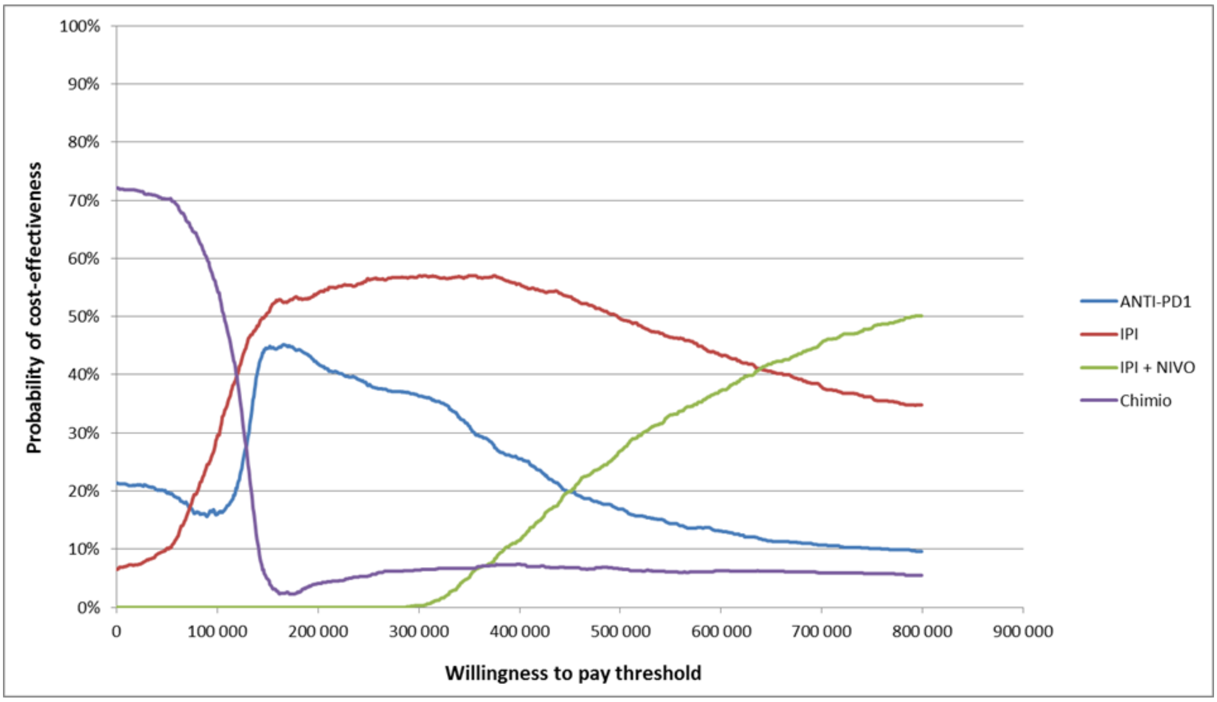

Figure S8: Cost-effectiveness acceptability curve for Wild-type population with a discount rate set at 0%

Table S1: Distribution of the baseline variables in BRAF-mutated population before weighting

Baseline variables	Bi-TT//Anti-PD1	Anti-PD1//Bi-TT		Ipi+Nivo//Bi-TT			Mono-TT//Bi-TT				Mono-TT//Anti-PD1				
	%	%	p value vs Bi-TT// Anti-Pd1	%	p value vs Bi-TT// Anti-Pd1	p value vs Anti-Pd1// Bi-TT	%	p value vs Bi-TT// Anti-Pd1	p value vs Anti-Pd1// Bi-TT	p value vs Ipi+Nivo// Bi-TT	%	p value vs Bi-TT// Anti-Pd1	p value vs Anti-Pd1// Bi-TT	p value vs Ipi+Nivo// Bi-TT	p value vs Ipi+Nivo// Bi-TT
**Brain metastases**			0.11		0.01	0.13		0.35	0.42	0.04		0.02	0.61	0.2	0.14
Absence	0.755	0.893		1			0.822				0.929				
Présence	0.245	0.107		0			0.178				0.071				
**Liver metastases**			0.15		0.1	0.02		0.14	0.82	0.02		0.34	0.56	0.05	0.67
Absence	0.685	0.821		5			0.8				0.762				
Présence	0.315	0.179		0.5			0.2				0.238				
**Elevated LDH**			0.09		0.001	0.12		0.31	0.04	0.001		0.69	0.28	0.009	0.25
No	0.699	0.536		0.3			0.778				0.667				
Yes	0.301	0.464		0.7			0.222				0.333				
**ECOG**			0.55		0.88	0.77		0.03	0.21	0.2		0.48	0.81	0.65	0.38
0 (=0,1)	0.86	0.857		0.9			0.689				0.81				
1 (=2,3,4)	0.077	0.036		0.05			0.156				0.071				
Unknown	0.063	0.107		0.05			0.156				0.119				
**Metastatic status**			0.07		0.93	0.23		0.6	0.48	0.71		0.1	0.43	0.25	0.33
0 (IIIc, M1a, M1b)	0.28	0.5		0.25			0.356				0.405				
1 (M1c)	0.657	0.464		0.7			0.6				0.476				
Unknown	0.063	0.036		0.05			0.044				0.119				
**High neutrophil to lymphocyte ratio**			0.06		0.91	0.05		0.24	0.3	0.21		0.14	0.46	0.13	0.74
Yes	0.622	0.857		0.6			0.756				0.786				
No	0.371	0.143		0.4			0.244				0.214				
**Mutation V600E**			0.87		0.09	0.02		0.75	0.53	0.06		0.006	0.7	0.001	0.006
Yes	0.189	0.214		0			0.156				0.429				
No	0.804	0.786		1			0.844				0.571				
**Age**			0.51		0.11	0.08		0.95	0.61	0.14		0.96	0.62	0.14	1
<65	0.671	0.607		0.85			0.667				0.667				
>65	0.329	0.393		0.15			0.333				0.333				
**Anatomic location**			0.52		0.67	NA		0.89	0.35	NA		0.71	0.54	0.46	0.62
Upper and lower extremities	0.259	0.286		0.15			0.2				0.286				
Trunk	0.441	0.464		0.55			0.444				0.333				
Head and neck	0.119	0.179		0.2			0.156				0.143				
Acral lentiginous	0.021	0		0			0.022				0.024				
Mucosa	0.014	0.036		0			0				0.048				
Unknown	0.147	0.036		0.1			0.178				0.167				
**BMI**			**0.57**		0.91	0.61		0.87	0.54	1		0.5	1	0.57	0.49
<18,5	0.811	0.857		0.8			0.8				0.857				
18.5<=BMI<25.0	0.189	0.143		0.2			0.2				0.143				

Table S2: Distribution of baseline variables in BRAF-mutated population after weighting

	Bi-TT//Anti-PD1	Anti-PD1//Bi-TT		Ipi+Nivo//Bi-TT			Mono-TT//Bi-TT				Mono-TT//Anti-PD1				
Baseline variables	%	%	p value vs Bi-TT// Anti-Pd1	%	p value vs Bi-TT// Anti-Pd1	p value vs Anti-Pd1// Bi-TT	%	p value vs Bi-TT// Anti-Pd1	p value vs Anti-Pd1// Bi-TT	p value vs Ipi+Nivo// Bi-TT	%	p value vs Bi-TT// Anti-Pd1	p value vs Anti-Pd1// Bi-TT	p value vs Ipi+Nivo// Bi-TT	p value vs Ipi+Nivo// Bi-TT
**Brain metastases**			0.92		**0.05**	0.36		0.47	0.68	0.32		0.54	0.59	**0.12**	0.32
Absence	0.783	0.796		1			0.851				0.685				
Présence	0.217	0.204		0			0.149				0.315				
**Liver metastases**			0.78		0.42	0.43		0.63	0.57	0.83		0.15	0.49	**0.09**	0.17
Absence	0.711	0.748		0.608			0.647				0.836				
Présence	0.289	0.252		0.392			0.353				0.164				
**Elevated LDH**			0.31		**0.09**	0.57		0.42	0.16	**0.05**		0.65	0.25	**0.09**	0.78
No	0.728	0.603		0.495			0.803				0.772				
Yes	0.272	0.397		0.505			0.197				0.228				
**ECOG**			0.58		0.26	0.1		0.52	0.98	**0.08**		0.86	0.69	0.32	0.69
0 (=0,1)	0.868	0.765		0.957			0.792				0.864				
1 (=2,3,4)	0.074	0.113		0.023			0.104				0.059				
Unknown	0.058	0.122		0.02			0.104				0.077				
**Metastatic status**			0.96		0.56	0.74		0.86	0.97	0.83		0.83	0.94	0.5	0.79
0 (IIIc, M1a, M1b)	0.302	0.286		0.18			0.253				0.333				
1 (M1c)	0.645	0.645		0.761			0.679				0.596				
Unknown															
**High neutrophil to lymphocyte ratio**			0.43		0.87	0.63		0.8	0.44	0.75		**0.09**	0.47	0.15	**0.06**
Yes	0.691	0.813		0.741			0.696				0.887				
No	0.298	0.187		0.259			0.304				0.113				
**Mutation V600E**			0.24		0.26	0.18		0.24	0.96	0.11		**0.02**	0.4	**0.007**	0.43
Yes	0.167	0.316		0			0.325				0.481				
No	0.822	0.684		1			0.675				0.519				
**Age**			0.61		**0.08**	**0.07**		0.36	0.77	**0.03**		0.29	0.24	0.41	0.13
<65	0.667	0.602		0.867			0.552				0.771				
>65	0.333	0.398		0.133			0.448				0.229				
**Anatomic location**			0.27		0.75	NA		0.43	0.38	NA		0.93	0.43	0.75	0.36
Upper and lower extremiti	0.252	0.365		0.177			0.292				0.224				
Trunk	0.46	0.371		0.608			0.319				0.533				
Head and neck	0.105	0.083		0.147			0.081				0.072				
Acral lentiginous	0.019	0		0			0.016				0.011				
Mucosa	0.014	0.113		0			0				0.019				
Unknown	0.149	0.069		0.069			0.293				0.14				
**BMI**			0.63		0.97	0.69		0.16	0.54	0.28		0.49	0.38	0.64	0.1
<18,5	0.829	0.775		0.833			0.667				0.879				
18.5<=BMI<25.0	0.171	0.225		0.167			0.333				0.121				

Table S3: Distribution of baseline variables in BRAF Wild-type population before weighting

	Anti-PD1	Ipilimumab		Ipilimumab + Nivolumab			Chimiothérapie			
Baseline variables	%	%	p value VS Anti-PD1	%	p value VS Anti-PD1	p value VS Ipilimumab	%	p value VS Anti-PD1	p value VS Ipilimumab	p value VS Ipilimumab +Nivolumab
**Brain metastases**			0.32		**0.06**	0.3		0.32	0.11	**0.02**
Absence	0.84	0.87		0.911			0.795			
Présence	0.16	0.129		0.089			0.205			
**Liver metastases**			0.76		**0.002**	**0.01**		0.42	0.61	0.12
Absence	0.76	0.753		0.616			0.723			
Présence	0.24	0.247		0.384			0.277			
**Elevated LDH**			0.64		0.14	**0.07**		0.83	0.8	0.21
No	0.712	0.726		0.625			0.711			
Yes	0.284	0.274		0.375			0.289			
**ECOG**			0.17		0.14	0.35		**0**	**0.001**	0.12
0 (=0,1)	0.827	0.828		0.786			0.663			
1 (=2,3,4)	0.059	0.054		0.098			0.193			
Unknown	0.114	0.118		0.116			0.145			
**Metastatic status**			0.1		0.68	**0.04**		0.13	**0.08**	0.17
0 (IIIc, M1a, M1b)	0.437	0.478		0.42			0.337			
1 (M1c)	0.519	0.511		0.518			0.639			
Unknown	0.043	0.011		0.062			0.024			
**High neutrophil to lymphocyte ratio**			0.43		0.6	0.92		**0.003**	**0.05**	**0.05**
Yes	0.743	0.72		0.741			0.59			
No	0.238	0.269		0.25			0.41			
**Age**			**0.006**		**0**	**0.07**		**0.04**	**0**	**0**
<65	0.305	0.419		0.527			0.193			
>65	0.695	0.581		0.473			0.807			
**Anatomic location**			**0.02**		0.69	**0.008**		0.6	0.26	0.36
Upper and lower extremities	0.286	0.344		0.241			0.386			
Trunk	0.194	0.194		0.214			0.193			
Head and neck	0.172	0.118		0.152			0.12			
Acral lentiginous	0.098	0.167		0.08			0.06			
Mucosa	0.113	0.059		0.17			0.096			
Unknown	0.128	0.118		0.134			0.145			
**BMI**			0.38		0.51	0.95		0.25	0.63	0.62
<18,5	0.775	0.806		0.804			0.831			
18.5<=BMI<25.0	0.225	0.194		0.196			0.169			

Table A4: Distribution of the baseline variables in BRAF-Wild-type population after weighting

	**Anti-PD1**	**Ipilimumab**		**Ipilimumab + Nivolumab**			**Chimiothérapie**			
Baseline variables	**%**	**%**	**p value VS Anti-PD1**	**%**	**p value VS Anti-PD1**	**p value VS Ipilumumab**	**%**	**p value VS Anti-PD1**	**p value VS Ipilumumab**	**p value VS Ipilumumab + Nivolumab**
**Brain metastases**			0.54		0.21	0.54		0.2	0.64	0.82
Absence	0.868	0.893		0.919			0.91			
Présence	0.132	0.107		0.081			0.09			
**Liver metastases**			0.26		0.61	0.65		0.93	0.4	0.67
Absence	0.75	0.805		0.778			0.744			
Présence	0.25	0.195		0.222			0.256			
**Elevated LDH**			0.37		0.65	0.71		0.36	0.83	0.6
No	0.71	0.764		0.736			0.78			
Yes	0.286	0.236		0.264			0.22			
**ECOG**			0.54		0.41	0.2		0.77	0.34	0.63
0 (=0,1)	0.827	0.796		0.878			0.838			
1 (=2,3,4)	0.059	0.041		0.048			0.069			
Unknown	0.114	0.163		0.073			0.092			
**Metastatic status**			0.26		0.92	0.31		0.39	0.55	0.44
0 (IIIc, M1a, M1b)	0.47	0.548		0.453			0.466			
1 (M1c)	0.493	0.443		0.503			0.525			
Unknown	0.037	0.01		0.044			0.009			
**High neutrophil to lymphocyte ratio**			0.49		0.35	0.61		0.13	0.14	**0.09**
Yes	0.743	0.781		0.821			0.673			
No	0.238	0.211		0.164			0.327			
**Age**			0.52		0.74	0.44		0.25	0.16	0.44
<65	0.337	0.387		0.315			0.245			
>65	0.663	0.613		0.685			0.755			
**Anatomic location**			**0.01**		0.84	0.06		0.82	0.16	0.99
Upper and lower extremities	0.286	0.284		0.352			0.337			
Trunk	0.194	0.18		0.199			0.24			
Head and neck	0.172	0.1		0.141			0.112			
Acral lentiginous	0.098	0.277		0.08			0.081			
Mucosa	0.113	0.059		0.135			0.128			
Unknown	0.128	0.101		0.085			0.102			
**BMI**			0.42		0.47	0.99		0.8	0.48	0.5
<18,5	0.794	0.829		0.83			0.776			
18.5<=BMI<25.0	0.206	0.171		0.17			0.224			

Table S5: Extrapolated life expectancy and cost per patient from the multistate model (MSM) at 5 years

	Sequence	Mean time spent in 1st line ([$CI_{95\%}$], (month, years))	Mean time spent in subsequent lines ([$CI_{95\%}$], (month, years))	Mean survival time ([$CI_{95\%}$], (month, years))	Mean QALYs [3] [$CI_{95\%}$]	Mean total cost per patient [$CI_{95\%}$], (€)	Sequence
**BRAF mutated Population**	**Mono-TT[1] → Anti-PD1**	19,1 [12.4-29.7]<br>1,6 [1.0-2.5]	11,9 [4.9-21.8]<br>1.0 [0.4-1.8]	31,0 [22.4-43.1]<br>2,6 [1.9-3.6]	1,9 [1.4-2.7]	287 438<br>[206 761-398 575]	28% [14-58]
	**Mono-TT[1]→ Bi-TT[2]**	11,1 [6.8-21.3]<br>0,9 [0.6-1.8]	19,9 [9.6-27.3]<br>1,7 [0.8-2.3]	31,0 [19.5-42.0]<br>2,6 [1.6-3.5]	1,8 [1.1-2.4]	331 755<br>[212 458-460 177]	29% [11-56]
	**Bi-TT[2]→Anti-PD1**	18,6 [13.3-25.4]<br>1,5 [1.1-2.1]	11,7 [5.6-16.8]<br>1.0 [0.5-1.4]	30.2 [22.6-37.0]<br>2,5 [1.9-3.1]	1,9 [1.4-2.3]	359 067<br>[273 022-446 768]	26% [13-43]
	**Anti-PD1→ Bi-TT[2]**	14,1 [5.6-21.5]<br>1.2 [0.5-1.8]	21,3 [10.0-33.7]<br>1,8 [0.8-2.8]	35,5 [21.4-45.8]<br>3.0 [1.8-3.8]	2,3 [1.4-3.0]	364 497<br>[217 350-476 339]	39% [14-70]
	**IPI+NIVO→ Bi-TT[2]**	9,2 [4.4-14.8]<br>0,8 [0.4-1.2]	18,1 [9.8-28.0]<br>1,5 [0.8-2.3]	27,3 [17.5-37.3]<br>2,3 [1.5-3.1]	1,6 [1.0-2.2]	461 595<br>[293 897-626 081]	25% [11-47]
**BRAF wild-type Population**	**Chemotherapy**	3,9 [2.1-6.4]<br>0,3 [0.2-0.5]	13,0 [7.1-20.1]<br>1,1 [0.6-1.7]	16,9 [10.9-24.4]<br>1,4 [0.9-2.0]	0,9 [0.6-1.3]	123 932<br>[76 828-182 857]	11% [4-25]
	**Anti-PD1**	12,0 [9.5-15.4]<br>1,0 [0.8-1.3]	6,5 [4.0-8.6]<br>0,5 [0.3-0.7]	18,5 [15.1-22.4]<br>1,5 [1.3-1.9]	1,1 [0.9-1.3]	146 156<br>[118 664-175 638]	11% [7-18]
	**Ipilimumab**	5,8 [3.9-8.1]<br>0,5 [0.3-0.7]	14,2 [8.4-19.7]<br>1,2 [0.7-1.6]	20,0 [14.1-25.3]<br>1,7 [1.2-2.1]	1,2 [0.8-1.5]	171 109<br>[126 278-211 202]	15% [7-26]
	**Ipi + Nivo**	18,0 [10.5-24.8]<br>1,5 [0.9-2.1]	4,6 [2.2-8.5]<br>0,4 [0.2-0.7]	22,5 [14.9-29.6]<br>1,9 [1.2-2.5]	1,4 [0.9-1.8]	298 803<br>[196 880-391 788]	16% [7-29]

[1]Mono-TT=Mono-targeted therapy, [2]Bi-TT=Bi-targeted therapy, CI= Confidence Interval, [3]QALY= Quality adjusted life yea

Table S6: Extrapolated life expectancy and cost per patient from the multistate model (MSM) at 15 years

	Sequence	Mean time spent in 1st line ([CI95%], (month, years))	Mean time spent in subsequent lines ([CI95%], (month, years))	Mean survival time ([CI95%], (month, years))	Mean QALYs [3] [CI95%]	Mean total cost per patient [CI95%], (€)
BRAF-mutated Population	**Mono-TT[1] → Anti-PD1**	23,1 [13.0-42.7]<br>1.9 [1.1-3.6]	22,5 [7.1-45.9]<br>1,9 [0.6-3.8]	45,6 [27.5-76.7]<br>3.8 [2.3-6.4]	2.8 [1.7-4.7]	425 674<br>[255 293-718 525]
	**Mono-TT[1] → Bi-TT[2]**	12,8 [6.9-27.1]<br>1,1 [0.6-2.3]	33,2 [13.4-56.7]<br>2,8 [1.1-4.7]	46,0 [24.0-74.8]<br>3,8 [2.0-6.2]	2,6 [1.4-4.3]	481 282<br>[258 297-781 441]
	**Bi-TT[2] → Anti-PD1**	21,7 [14.4-34.8]<br>1,8 [1.2-2.9]	19,8 [8.0-32.6]<br>1,7 [0.7-2.7]	41,5 [27.1-59.0]<br>3,5 [2.3-4.9]	2,6 [1.7-3.7]	476 134<br>[324 434-678 893]
	**Anti-PD1 → Bi-TT[2]**	16,5 [5.6-27.3]<br>1,4 [0.5-2.2]	39,2 [14.9-76.7]<br>3,3 [1.2-6.4]	55,7 [27.8-91.3]<br>4,6 [2.3-7.6]	3,7 [1.8-6.1]	589 511<br>[283 308-998 899]
	**IPI+NIVO → Bi-TT[2]**	9.7 [4.5-17.0]<br>0.8 [0.4-1.4]	29,7 [14.5-54.2]<br>2,5 [1.2-4.5]	39,4 [22.4-65.0]<br>3,3 [1.9-5.4]	2,3 [1.3-3.8]	601 745<br>[362 953-919 625]
BRAF wild-Type Population	**Chemotherapy**	4,1 [2.2-6.9]<br>0,3 [0.2-0.6]	17,7 [8.3-30.6]<br>1,5 [0.7-2.6]	21,9 [12.4-34.7]<br>1,8 [1.0-2.9]	1,2 [0.7-1.9]	163 284<br>[86 085-267 023]
	**Anti-PD1**	14,2 [10.8-19.1]<br>1,2 [0.9-1.6]	9,3 [5.1-13.4]<br>0,8 [0.4-1.1]	23,5 [17.4-29.5]<br>2,0 [1.5-2.5]	1,4 [1.0-1.7]	186 435<br>[138 048-233 660]
	**Ipilimumab**	5,9 [4.0-8.7]<br>0,5 [0.3-0.7]	20,2 [10.4-30.4]<br>1,7 [0.9-2.5]	26,0 [16.4-36.2]<br>2,2 [1.4-3.0]	1,5 [1.0-2.1]	203 528<br>[143 342-265 906]
	**Ipi + Nivo**	22,9 [11.8-35.4]<br>1,9 [1.0-2.9]	5,9 [2.7-13.7]<br>0,5 [0.2-1.1]	28,8 [17.6-43.2]<br>2,4 [1.5-3.6]	1,8 [1.1-2.6]	381 807<br>[238 165-571 985]

[1]Mono-TT=Mono-targeted therapy, [2]Bi-TT=Bi-targeted therapy, CI= Confidence Interval, [3]QALY= Quality adjusted life years

Table S7: Extrapolated life expectancy and cost per patient from the multistate model (MSM) with a discount rate set at 0%

	Sequence	Mean time spent in 1st line ([$CI_{95\%}$], (month, years))	Mean time spent in subsequent lines ([$CI_{95\%}$], (month, years))	Mean survival time ([$CI_{95\%}$], (month, years))	Mean QALYs [3] [$CI_{95\%}$]	Mean total cost per patient [$CI_{95\%}$], (€)
**BRAF-mutated Population**	**Mono-TT[1] → Anti-PD1**	23,3 [13.9-43.6]<br>1,9 [1.2-3.6]	22,1 [7.9-44.8]<br>1,8 [0.7-3.7]	45,4 [28.6-75.6]<br>3,8 [2.4-6.3]	2.8 [1.8-4.7]	423 807<br>[263 409-705 367]
	**Mono-TT[1]→ Bi-TT[2]**	12,8 [7.2-28.3]<br>1,1 [0.6-2.4]	34,2 [13.8-54.0]<br>2,8 [1.2-4.5]	47,0 [25.2-72.6]<br>3,9 [2.1-6.0]	2,7 [1.4-4.2]	491 321<br>[267 755-767 535]
	**Bi-TT[2]→Anti-PD1**	23,4 [15.0-35.7]<br>2,0 [1.2-3.0]	19,8 [8.3-31.3]<br>1,6 [0.7-2.6]	43,2 [28.6-59.3]<br>3,6 [2.4-4.9]	2,7 [1.8-3.7]	498 928<br>[342 620-693 808]
	**Anti-PD1→ Bi-TT[2]**	16,7 [5.8-28.5]<br>1,4 [0.5-2.4]	38,0 [15.4-71.7]<br>3,2 [1.3-6.0]	54,8 [28.4-86.8]<br>4,6 [2.4-7.2]	3,6 [1.9-5.8]	577 664<br>[292 432-936 186]
	**IPI+NIVO→ Bi-TT[2]**	10,2 [4.5-17.8]<br>0,8 [0.4-1.5]	29,7 [14.9-53.0]<br>2,5 [1.2-4.4]	39,9 [22.8-63.7]<br>3,3 [1.9-5.3]	2,3 [1.3-3.7]	616 427<br>[372 412-915 269]
**BRAF Wild-Type Population**	**Chemotherapy**	4,2 [2.2-7.1]<br>0,4 [0.2-0.6]	18,3 [8.7-31.1]<br>1,5 [0.7-2.6]	22,5 [12.7-35.2]<br>1,9 [1.1-2.9]	1,2 [0.7-1.9]	168 043<br>[90 235-273 329]
	**Anti-PD1**	15,9 [11.3-19.6]<br>1,3 [0.9-1.6]	9,1 [5.4-13.9]<br>0,8 [0.5-1.2]	25,0 [18.5-30.6]<br>2,1 [1.5-2.5]	1,5 [1.1-1.8]	197 155<br>[145 058-243 952]
	**Ipilimumab**	5,7 [4.0-9.1]<br>0,5 [0.3-0.8]	21,0 [11.0-31.4]<br>1,7 [0.9-2.6]	26,7 [17.2-37.2]<br>2,2 [1.4-3.1]	1,6 [1.0-2.2]	205 639<br>[1466 996-272 755]
	**Ipi + Nivo**	23,4 [12.4-36.0]<br>2,0 [1.0-3.0]	6,0 [2.9-14.0]<br>0,5 [0.2-1.2]	29,4 [17.9-44.0]<br>2,4 [1.5-3.7]	1,8 [1.1-2.7]	389 178<br>[243 483-587 907]

[1]Mono-TT=Mono-targeted therapy, [2]Bi-TT=Bi-targeted therapy, CI= Confidence Interval, [3]QALY= Quality adjusted life years
